# Secondary Prevention in Radiologically Isolated Syndromes and Prodromal Stages of Multiple Sclerosis

**DOI:** 10.3389/fneur.2022.787160

**Published:** 2022-03-14

**Authors:** Maria Pia Amato, Nicola De Stefano, Matilde Inglese, Emanuele Morena, Giovanni Ristori, Marco Salvetti, Maria Trojano

**Affiliations:** ^1^Department of Neurosciences, Psycology, Drug Research and Child Health (NEUROFARBA), University of Florence, Florence, Italy; ^2^Fondazione Don Carlo Gnocchi, Florence, Italy; ^3^Department of Medicine, Surgery and Neuroscience, University of Siena, Siena, Italy; ^4^Department of Neuroscience, Rehabilitation, Ophthalmology, Genetics, Maternal and Child Health (DINOGMI), University of Genoa, Genoa, Italy; ^5^San Martino Hospital-IRCCS, Genoa, Italy; ^6^Department of Neuroscience, Mental Health and Sensory Organs (NESMOS), Sapienza University, Rome, Italy; ^7^Neuroimmunology Unit, IRCCS Fondazione Santa Lucia, Rome, Italy; ^8^Istituto di Ricovero e Cura a Carattere Scientifico (IRCCS) Istituto Neurologico Mediterraneo (INM) Neuromed, Pozzilli, Italy; ^9^Department of Basic Medical Sciences, Neurosciences and Sense Organs, University of Bari Aldo Moro, Bari, Italy

**Keywords:** clinically silent demyelination, radiologically isolated syndrome, prodromal multiple sclerosis, endophenotype, preventive approaches clinically silent demyelination, BCG—Bacille Calmette-Guérin vaccine, vaccine, preventive approach

## Abstract

Following the extraordinary progress in the treatment of multiple sclerosis (MS), two major unmet needs remain: understanding the etiology of the disease and, hence, designing definitive cures (this perspective is neither at hand, nor it can be taken for granted that the etiologic targets will be readily treatable); the prevention of an overt and disabling disease, which seems to be a more realistic and pragmatic perspective, as the integration of genetic data with endophenotypes, MRI, and other biomarkers ameliorates our ability to identify early neuroinflammation. Radiologically isolated syndrome (RIS; diagnosed when the unanticipated MRI finding of brain spatial dissemination of focal white matter lesions highly suggestive of MS occurs in subjects without symptoms of MS, and with normal neurological examinations) and the recently focused “prodromal MS” are conditions at risk of conversion toward overt disease. Here, we explore the possibility of secondary prevention approaches in these early stages of neuroinflammation. RIS and prodromal MS are rare conditions, which suggest the importance of Study Groups and Disease Registry to implement informative clinical trials. We summarize ongoing preventive approaches in the early stages of the demyelinating process, especially in RIS conditions. Moreover, we highlight the importance of the biomarkers and the predictors of evolution to overt disease, which may be useful to select the individuals at risk of conversion to clinically isolated syndrome (CIS) and/or clinically definite MS. Finally, we illustrate the importance of the endophenotypes to test the frontline immunomodulatory approach for preventive strategies. Future investigations, especially in relatives of patients, based on MRI techniques and biological studies (better with integrated approaches) may provide opportunities to understand the MS early causal cascade and may help to identify a “therapeutic window” to potentially reverse early disease processes.

## Introduction

The occurrence of incidental brain white matter lesions suggestive of multiple sclerosis (MS) in subjects who did not have symptoms or signs of MS during their lifetime is well-documented, as it was described several decades ago in several postmortem studies. The widespread use of MRI, as the standard *in vivo* study of central nervous system (CNS) demyelination, has greatly increased the detection of the asymptomatic brain and spinal cord abnormalities of uncertain clinical significance. In 2009, Okuda et al. neologized formally this entity, using the term radiologically isolated syndrome (RIS) (1). More recently, the concept of prodrome (an early set of signs, symptoms, or other findings that occur before the onset of the typical disease features) has begun to be considered in MS (2), thanks to several investigations based on population-based studies and biomarkers of early CNS damage (refer below).

The present review will focus on these conditions potentially leading to overt MS, to consider current attempts of secondary prevention, in the absence of an etiologic therapy. The possible integration of genetic data with endophenotypes, MRI data, and other biomarkers seems to promise fruitful approaches to the aim of counteracting the development of the overt disease. To provide a survey on these topics, we searched PubMed for all articles published from database inception to September 1, 2021, with no language limitations. Keywords included clinically silent demyelination, prodromal MS, RIS, subclinical MS, endophenotype of MS, MS prevention.

## Clinically Silent Demyelination

Neuropathological studies demonstrated that brain demyelination might remain clinically silent for the whole lifetime in a significant proportion of people (about 0.1–0.3% of the autopsies in those studies) (1). The location of lesions in clinically silent areas, the low degree of inflammation, or even a particularly effective individual response to injury (e.g., functional compensatory adaptation, neuronal plasticity, and repair) might explain the absence of clinically relevant signs of MS in those subjects. However, caution is needed when interpreting these data, as it is difficult to ascertain whether these subjects were truly asymptomatic with normal neurological examination during their life (3). A recent study demonstrated, for example, that 33% of patients consulting for a first demyelinating event had prior symptoms suggestive of central nervous system (CNS) demyelination that had gone unnoticed (4). Moreover, the samples included in the studies were not representative of the general population due to selection bias toward those who were subjected to autopsy. Finally, such figures are probably underestimated nowadays, in view of an increasing prevalence and incidence of MS.

The advent of MRI and its development as the most sensitive and prominent paraclinical tool for the evaluation of morphologic brain abnormalities has modified our perspective on the occurrence of incidental brain findings. In a large meta-analysis (more than 15,000 subjects from 16 studies), the prevalence of neoplastic and non-neoplastic incidental findings on brain MRI was 2.7%, with the observed incidence increasing with age (5). Among those, only <0.1% could be interpreted as inflammatory–demyelinating lesions if white matter hyperintensities of suspected cerebrovascular origin were excluded. Similar prevalence for an MRI pattern suggestive of MS was found in a recent study that performs a systematic revision of the MRI scans and related clinical charts (1,907 individuals) in a high-incidence region for MS (6). These figures are higher, however, in asymptomatic first-degree relatives of both patients with sporadic MS (4%) and families with members affected by MS (10%) (7). In a recent prospective population-based study, incidental findings on brain MRI necessitating further diagnostic evaluation, but mostly without direct clinical consequences, were found in over 3% of the general middle-aged and elderly population, although no case with demyelinating lesions was reported (8).

According to *ex vivo* and *in vivo* data, the occurrence of silent demyelination should be, therefore, considered uncommon in clinical practice, with a higher occurrence in specific conditions such as family members of patients with MS. However, it must be stressed that the growing use of MRI has significantly increased the probability to find asymptomatic intracranial abnormalities of potential clinical significance, which includes silent demyelination, in current prospective studies in comparison with previous retrospective or neuropathological studies.

## Emerging Evidence for a Prodromal Phase of MS

The precise etiology of MS is not yet known, although the evidence pertaining to different research fields indicates that genetic and environmental factors interact with each other in a complex manner, which eventually determines an abnormal autoimmune response (9). In particular, the evidence that environmental factors can play a role long before the clinical onset of MS is well-established and suggests the existence of a prodromal phase for the disease. The possibility of a prodrome indicates a window of opportunity to potentially act on early disease processes before the clinical disease becomes evident.

The concept of a prodrome is defined as the time period between the onset of a decline in a baseline level of functioning until criteria for disease diagnosis are met (10). The question of whether there is a prodrome in MS has not been extensively studied so far. Other neurodegenerative diseases, such as Parkinson's disease and Alzheimer's disease, and other inflammatory autoimmune diseases, such as rheumatoid arthritis and inflammatory bowel disease, in which several biomarkers are better established, have a more advanced understanding of their prodromal phase than we currently have in MS (11). However, the last 10 years have provided increasing evidence that also MS may have a prodromal phase. Recently, population-based studies have demonstrated that it is possible to objectively measure a symptomatic prodromal period in MS, which may last 5–10 years or perhaps long before the occurrence of classical “MS symptom onset.” In one case-control study conducted in Canada (12), the analysis of health administrative data linked with MS revealed that the use of healthcare services by patients was higher in the 5 years preceding their first clinical demyelinating event than that of controls. In the year before the first clinical demyelinating event, hospitalizations and physician visits were 78 and 88% higher, respectively, for people with MS than for matched controls. Similarly, dispensed prescription medications were 49% higher among patients who went on to develop MS. A subsequent case-control study conducted in the UK revealed a significantly higher number of visits to general practitioners among patients with MS, considering a time window up to 10 years before the first MS record (13).

Regarding the clinical profile of the prodromes, an elevated mental health burden is evident, with more visits to psychiatrists and diagnoses of depression and anxiety. Other issues include pain and headache, gastrointestinal complaints, bladder issues, sleep disturbances, and cognitive problems (11). Low cognitive performance before the onset of typical MS symptoms has been reported in a nested case-control study in Norway in men who entered the mandatory national military service at the age of 18–19 years (2). Moreover, fewer pregnancies and greater use of hormonal contraceptives have also been observed in the 5-year prodromal period, particularly in the year before MS onset, relative to a matched population without MS (12).

However, the possibility of identifying a prodromal syndrome exclusively on a clinical base has to be interpreted critically, as the above observations rely mostly on symptoms that are non-specific and common in the general population as well. To reliably identify a prodromal phase of MS, further research is needed that focuses on the identification of biomarkers. Indeed, biological markers of inflammation or neurodegeneration that indicate preexisting disease provide support to a pathogenic process underlying the prodromal period in MS: serum levels of neurofilament light chain (NfL) were increased up to 6 years before MS onset in 30 MS cases relative to 30 healthy controls (14). Such biomarkers are likely to be critical to distinguish whether or not non-specific symptoms such as fatigue represent the prodromal phase of neurodegenerative disease.

### Radiologically Isolated Syndrome as a Rare Condition: The Importance of Study Groups and Disease Registry

The radiologically isolated syndrome is a rare condition, although the exact prevalence of RIS is still unknown. One large hospital-based study in Sweden indicated a prevalence of 0.05% (0.15% among those aged 15–40 years) among 2,105 individuals who underwent MRI for any reason during a 1-year period (15). A meta-analysis that includes about 16,000 individuals with no history of neurological symptoms reported that 0.06% had MRI findings that were suggestive of demyelination (5). It is well-known that subjects with RIS can evolve toward relapsing-remitting (RR) or progressive (PP) MS, as in detail reported in the next paragraph.

**Figure 1:**
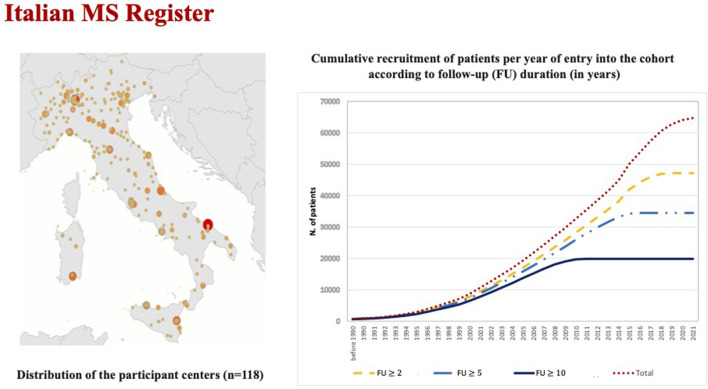
The figure on the right reports the increasing temporal trends of the total cohort and sub-cohorts with different follow-up duration (≥ 2.0 years: *n* = 47,161, ≥ 5.0 years: *n* = 34,488, and ≥ 10 years: n = 19,873).

The current available national and international MS databases and registries—“big MS data”—constitute the key tools to develop clinical research in the field of rare conditions, to improve healthcare planning and new clinical perspectives based on real-world data (16). By collecting longitudinal data on clinical and MRI disease activity over time, MS registries become crucial in the study of the natural history of patients with RIS, to assess the risk factors associated with the conversion to clinically definite MS, and to identify candidates for possible preventive or therapeutic approaches. MS clinical data sharing initiative has a longstanding tradition in Italy for over 20 years. In 2014, the Italian MS Foundation, in collaboration with the University of Bari, promoted the creation of the Italian MS Register (17). Currently, it is one of the largest registers in Europe, with 118 MS centers, that provided data of 72,202 patients (about 50,000 of them with a longitudinal follow-up >5 years) in different phases of the disease, which include RIS subject (Figure 1).

Registries can also offer the opportunity of including longitudinal evaluation of neuropsychological testing, quantitative MR metrics, and biological markers in subjects with RIS suggestive of MS. An Italian study on patients recruited from 5 MS centers highlighted that cognitive impairment of the same profile as that of RRMS was found in 27.6% of subjects with RIS, and comparable levels of MRI lesion loads and brain atrophy were found in RIS and RRMS (18). In a more recent analysis of prospectively collected data from a population-based registry of the MS Center of Tel Aviv (19), cognitive performance was relatively preserved in RIS subjects, although all cognitive measurements, in particular those related to information processing speed, were below the mean performance of age- and education-matched healthy population. The crucial assumption of this article was that the cognitive performance of RIS subjects should be followed closely to identify any changes that may indicate conversion to MS.

### Predictors of Evolution in RIS

There is an ever-growing effort for improving the characterization of RIS and identifying predictors of clinical and radiological evolution. This is important not only to prevent diagnosis but also to improve knowledge on prognosis and provide guidelines for surveillance or prophylactic treatment.

The most important risk factors for an initial clinical event have been identified by the collective effort of the Radiologically Isolated Syndrome Consortium (RISC), which led to two main reports related to the 5- and 10-year risk of developing a first clinical event. Specifically, the estimated 5-year risk of developing a first clinical event was 34% in a cohort of 451 RIS subjects (86% women) with a mean age at RIS diagnosis of 37.2 years. The independent predictors were male gender, young age (age ≤37 years), and the presence of MRI spinal cord lesions at baseline (20). Fifteen patients from the same cohort evolved to primary progressive MS (PPMS) in a median time of 3.5 years. Male patients with older age and a higher number of spinal cord lesions were at higher risk of PPMS evolution (21).

The cumulative probability of a first clinical event at 10 years was 51.2% in the same cohort. The independent predictors of a subsequent clinical event were age, the presence of cerebrospinal fluid (CSF)-restricted oligoclonal bands (OB), MRI infratentorial lesions, and spinal cord lesions at baseline and gadolinium-enhancing lesions during follow-up (22). The same group recently confirmed the predictors of 10-year conversion to MS and reported data relevant to the number of enrolled patients needed to detect a potential treatment effect (23).

Furthermore, a study conducted in an international historical cohort of 61 children with RIS who were followed longitudinally for a mean of 4.2 ± 4.7 years further enforced the importance of CSF OB whose presence increased the specificity of MRI criteria to predict MS in children with RIS (24).

A recent study employed optical coherence tomography (OCT), a non-invasive imaging technique that uses light waves to take cross-section pictures of the retina, to investigate whether it plays a role as a predictor of evolution in individuals with RIS. A total of 36 RIS subjects were followed up for a mean of 46 [26–58] months; the eight RIS subjects who converted to MS showed a thinning of the peripapillary retinal nerve fiber layer (pRNFL). Specifically, subjects with a pRNFL of 99 μm or lower were at a 7.5-fold risk for MS conversion compared to individuals with higher pRFNL measures. The Cox proportional hazards regression revealed a hazard ratio of 1.08 for conversion to MS for each 1 μm decline in pRNFL, which suggests that OCT might be useful for risk stratification in RIS subjects (25). Similar results were reported in a work showing that OCT can be potentially useful for predicting prognosis in RIS, being OCT measurements associated with brain volumetrics and clinical conversion to MS (26).

Among biomarkers explored as the potential predictors of RIS evolution, levels of CSF IL8, a marker of diffuse intrathecal inflammation, and CSF NfL levels have shown some promising results. A small study that includes 18 RIS subjects showed higher CSF IL8 levels in MS converters than in non-converters (*p* = 0.03). Moreover, in the multivariate regression model including known predictors such as age, gender, and the presence of spinal cord lesions, a high level of CSF IL8 was an independent predictor of MS conversion in RIS (*p* = 0.02) (27). A larger study investigated the prognostic role of chitinase 3-like 1 (CHI3L1), NfL, and OB for conversion to clinically isolated syndrome (CIS) and MS in 75 RIS subjects. In contrast to CHI3L1, which did not show any influence on clinical conversion, NfL levels and OB were the independent risk factors for the development of CIS and MS. Fixing the best cutoff at a CSF NfL level of 619 ng/l, higher values were associated with a trend to shorter time to CIS (*p* = 0.079) and a significantly shorter time to MS (*p* = 0.017), which supports the importance of CSF analysis in individuals with RIS (28).

The available data provide evidence that a meaningful number of RIS subjects evolve to MS and support the need for standardized biomarkers to identify those subjects at greatest risk for conversion to MS who need appropriate clinical and treatment management.

### Ongoing Preventive and Therapeutic Approaches in RIS

Following the extraordinary progress in the treatment of overt MS, a major unmet need remains for translational research: preventing or significantly slowing the disease onset or the progression of the neuroinflammatory process to aim at the “dream” of a world free of MS. We can hope to make the dream come true by understanding the etiology of the disease and hence designing definitive cures. Unfortunately, this perspective is neither at hand, nor it can be taken for granted that the etiologic targets, once discovered, will be readily treatable. A more realistic and pragmatic perspective may be the prevention of the clinical onset of the disease, a research field that promises to become increasingly important as the integration of genetic data with endophenotypes, MRI, and other biomarkers ameliorates our ability to act before the development of the overt disease (refer to next paragraph).

Radiologically isolated syndrome falls within the endophenotypes and thus offers the opportunity to try to prevent the onset of the clinical demyelinating disorder. The best approach to this aim remains an object of controversy. In particular, whether or not to treat the RIS remains currently a clinical conundrum: several recommendations and guidelines have been published (29–31). To summarize the various standpoints, the absence of clinical disturbances suggests caution against interventions; on the other hand, the presence of OB in the CSF or signals of progression at MRI (besides the above-reported predictors of conversion to clinical disease) prompt MS specialists to consider disease-modifying therapies (DMTs) presently used for CIS or MS.

Three therapeutic approaches are currently registered at ClinicalTrials.gov for RIS (whereas no trial comes out for “prodromal MS”): NCT03122652 with teriflunomide, NCT02739542 with dimethyl fumarate, and the recently proposed NCT04877457 with ocrelizumab. Epidemiological data support the view that vitamin D supplementation, prevention of metabolic disorders, and smoking avoidance are candidate approaches for primary and secondary prevention of MS (32). In a recent study, which shows the prevalence of RIS and white matter abnormalities in healthy relatives of patients with MS, smoking was associated with the presence of multiple altered signals in white matter and obesity with the fulfillment of RIS pattern (33). The study, together with evidence coming from numerous epidemiologic investigations in MS people, would suggest preventive attempts in an early condition such as RIS. However, rather unexpectedly, preventive approaches based on vitamin D supplementation, reduction of metabolic pressure by diet, and smoking avoidance are not currently tried in RIS; rather, ongoing registered trials are largely oriented toward DMT used in MS management (see above).

**Figure 2:**
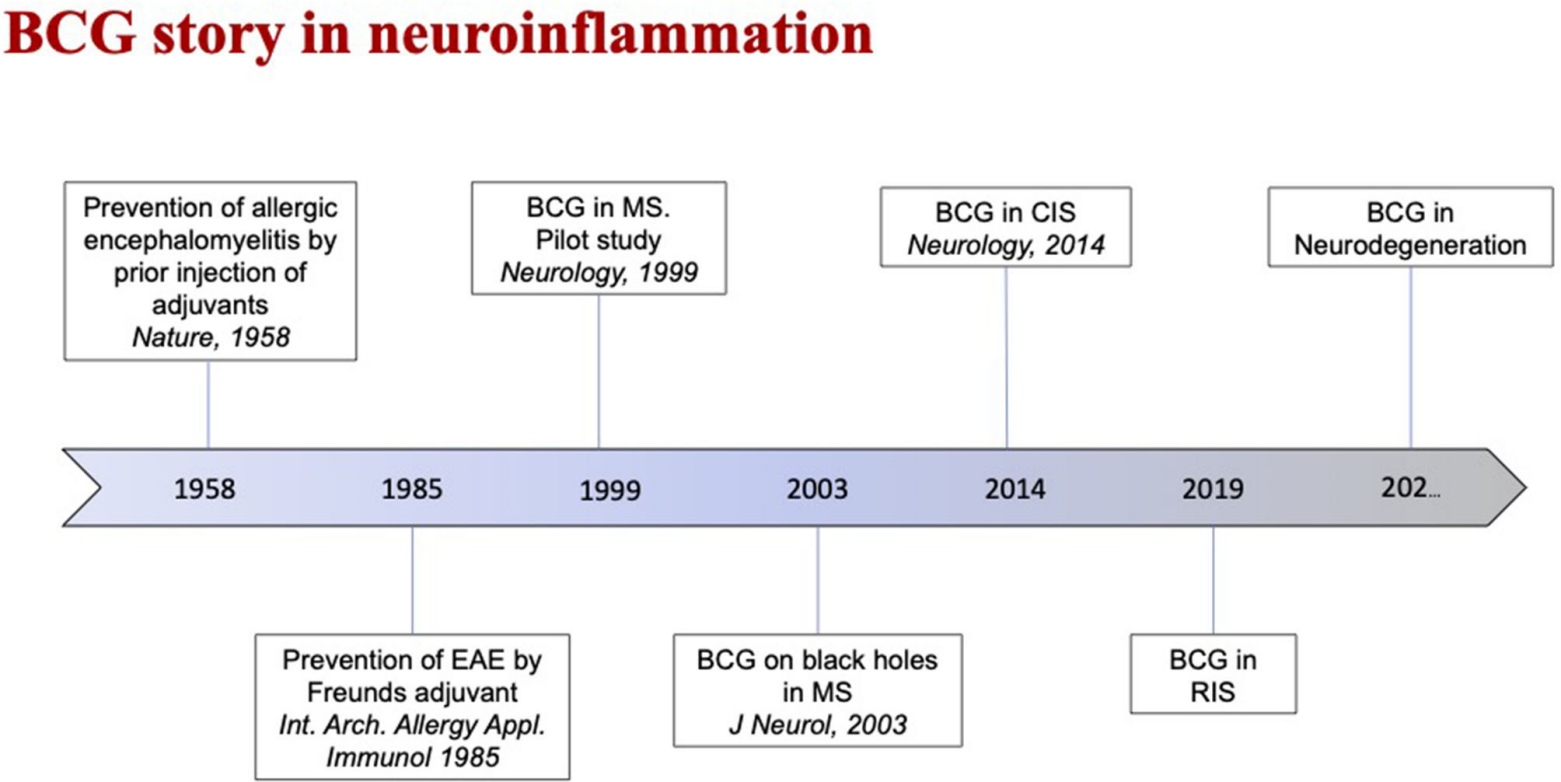
This time-line highlights the evolution of the role of Bacille Calmette-Guérin (BCG) vaccine in neuroinflammatory diseases as Multiple Sclerosis (MS).

Among other approaches that may have characteristics compatible with a preventive intervention, BCG vaccination has been tested with encouraging results in early MS and CIS (34–36). Italian groups proposed this approach as a sort of secondary prevention (rather than as a treatment) against RIS progression, also given the characteristics of the BCG vaccine, which is safe, cheap, and handy. A phase II, double-blind, randomized, controlled, multicenter study with two parallel groups of subjects (one arm will be vaccinated with a single dose of BCG vaccine and the other with placebo) is currently ongoing (NCT03888924). The rationale includes, among the others, the fact that the BCG vaccine may prevent the development of neuroinflammation by antagonizing the effects of “Westernization.” This approach could in fact somehow compensate for the deprivation of benign exposure to microbes and the changes of lifestyle habits that occurred over the last decades of the twentieth century in developed countries. The “Westernization” seems to be associated with the increased incidence of complex diseases, such as cancer, metabolic disorders, and neurodegenerative diseases, and immunopathological conditions, such as autoimmune and atopic disorders (37–39). Recent evidence on the possible preventive effects of the BCG vaccine against the development of neurodegenerative diseases (such as Alzheimer's and Parkinson's diseases, which are supposed not to be of primary inflammatory nature; Figure 2) contribute to support this view (40–43).

## Incidental MRI Findings or Subclinical MS? The Contribution of Biological Endophenotypes

Since its first description, RIS has been widely debated and the risk of RIS evolving into MS has been investigated. According to existing data (20, 22), several RIS subjects evolve to MS over time, which demonstrates that RIS, at least in some cases, represents a preclinical stage of MS (refer to the paragraph “predictors of evolution in RIS”). The first issue related to this lies in what still needs to be done to provide a more specific characterization of these asymptomatic subjects and accurately discriminate subjects who can have a subclinical form of MS. In this context, assuming a carefully collected clinical history and a meticulous clinical examination, the first step of the management of these asymptomatic subjects is to consider an appropriate differential diagnosis and assess the extent to which MRI lesions fulfilling the RIS criteria in asymptomatic subjects may be related to disorders other than MS. Moreover, the reason for the initial brain MRI should always be carefully considered. Whereas, there are subjects whose brain MRI is performed for reasons which have no relation with CNS or MS, on many occasions, MRI is performed due to symptoms that might be somehow related to MS. Headache is by far the most common reason for performing an MRI (about 50% of cases with RIS; 20), but other relatively less frequent indications for an MRI are also seizures, paroxysmal symptoms, anxiety, depression, and other psychiatric disorders. Whereas, it is not possible to establish whether these conditions are related to the MRI findings, it is also true that they might represent unusual clinical symptoms associated with MS (refer above: prodromal MS).

Another important contribution may come from laboratory studies, which start to delineate biological signatures capable of integrating MRI data to refine the condition of subclinical neuroinflammation. The potential utility of the NfL levels was already reported in the context of the prodromal MS and RIS (refer above). However, this biomarker, though sensitive and able to peripherally mirror CNS tissue damage, is not specific, as demonstrated by studies in other CNS diseases, especially of primary neurodegenerative nature (44).

As anticipated according to MRI data, studies in relatives of MS patients are especially informative to identify MS endophenotypes. A recent system biology approach on peripheral immune signatures in identical twin pairs discordant for MS showed remarkably similar patterns; however, distinct traits in effector CD4+ T-cells in clinically healthy twins, with signs of prodromal MS, were comparable with those of the overtly affected co-twins, suggesting the importance of these immune traits in subclinical neuroinflammation (45). On the same line, increased CSF sulfatide levels and serum autoantibody against glycosphingolipids were reported in healthy siblings of patients MS compared to unrelated healthy donors (46).

Concerning the identification of endophenotypes due to genetic risk, glutamate biology seems to contain relevant biomarkers that pose a risk for disease development. Associations of at-risk single nucleotide polymorphisms (SNPs) with high glutamate concentration in CNS (47) or with brain volume changes in MS (48) were described, and they may contribute to clarifying the MS genetic risk in the “target” organ. Recent development in multiomics approaches have demonstrated alterations in easily accessible fluids: correlating scores of genetic risk and blood analytes, Wainberg et al. showed changes in clinically healthy individuals that mirrored those seen in people with complex diseases, including MS, and that represented early signs of dysfunctions preceding the clinically overt disease (49). Considering the infectious mononucleosis (IM) as a non-genetic risk factor for MS, Jons et al. assayed MS-relevant CSF cyto- or chemokines from non-MS individuals with or without previous IM and MS people as a reference group. They found a stepwise inflammation from IM sequelae to an MS endophenotype in a subgroup of IM patients, which shows CSF changes comparable to those of the MS reference group (50).

## Conclusion

Extreme caution is needed in classifying RIS subjects. Indeed, it should be stressed that only when these subjects are expertly diagnosed, the stratification of risk can be accurate, and we can thus have sufficient information to be able to differentiate subjects with a form of subclinical MS at low or high risk of developing the disease (22, 23, 29).

Even more, caution is needed to identify a prodromal MS syndrome, which is largely based on symptoms that are non-specific and common in the general population, and that currently lacks objective supports to disentangle a “real” condition preluding to MS. Future advances in MRI techniques, biological studies, and especially integrated approaches to identify and follow individuals at high disease risk are a concrete hope for the identification of the initial phases of the demyelinating process and better MS management. This kind of investigation may provide a powerful opportunity to understand the MS early causal cascade, and, more importantly, may help to identify a “therapeutic window” to potentially reverse early disease processes.

## Author Contributions

MPA has focused on prodromes, predictors of evolution, and silent demyelination. NDS contributed describing prodromal phase of MS and silent demyelination. MI reported the role of predictors of evolution in RIS. EM, GR, and MS contributed in treatment and biological endophenotypes description. MT has focused on Study Groups and Disease Registry information. All authors contributed to the article and approved the submitted version.

## Conflict of Interest

The authors declare that the research was conducted in the absence of any commercial or financial relationships that could be construed as a potential conflict of interest.

## Publisher's Note

All claims expressed in this article are solely those of the authors and do not necessarily represent those of their affiliated organizations, or those of the publisher, the editors and the reviewers. Any product that may be evaluated in this article, or claim that may be made by its manufacturer, is not guaranteed or endorsed by the publisher.
